# PROTOCOL: Health, social care and technological interventions to improve functional ability of older adults: Evidence and gap map

**DOI:** 10.1002/cl2.1054

**Published:** 2019-10-10

**Authors:** Vivian Welch, Tracey E. Howe, Sue Marcus, Christine M. Mathew, Ritu Sadana, Morwenna Rogers, Lisa Sheehy, Johan Borg, Kevin Pottie, Joanna Thompson‐Coon, Anne Lyddiatt, Elizabeth Kristjansson, Jason W. Nickerson, Peter Walker, Peter Tanuseputro, Beverly Shea, Heidi Sveistrup, Panteha Babelmorad, Wei Zhang

**Affiliations:** ^1^ Methods Centre Bruyère Research Institute Ottawa Canada; ^2^ School of Health & Life Sciences Glasgow Caledonian University Glasgow UK; ^3^ Radcliffe Department of Medicine University of Oxford Oxford UK; ^4^ Bruyère Research Institute Ottawa ON Canada; ^5^ Ageing and Life‐course World Health Organization Geneva Switzerland; ^6^ NIHR PenCLAHRC, Institute of Health Research University of Exeter Medical School Exeter UK; ^7^ Division of Social Medicine and Global Health Lund University Malmo Sweden; ^8^ Department of Family Medicine University of Ottawa Ottawa ON Canada; ^9^ NIHR CLAHRC South West Peninsula (PenCLAHRC) University of Exeter Medical School Exeter UK; ^10^ Ingersoll ON Canada; ^11^ School of Psychology, Faculty of Social Sciences University of Ottawa Ottawa Canada; ^12^ Department of Medicine The Ottawa Hospital Ottawa ON Canada; ^13^ Ottawa Hospital Research Institute Ottawa ON Canada; ^14^ Vaccines and Health Products World Health Organization Geneva Switzerland; ^15^ School of Rehabilitation Sciences, Faculty of Health Sciences University of Ottawa Ottawa ON Canada

## Abstract

This is a protocol for a Campbell Evidence and Gap Map. The objectives are to identify and assess the available evidence on health, social care and technological interventions to improve functional ability among older adults.

## 1 BACKGROUND

### 1.1 Introduction

#### 1.1.1 The problem, condition or issue

There is an increasing proportion of older adults in the global population, with UN population projections predicting that before 2020, people aged >65 years will outnumber children aged <5 years for the first time in history (United, 2017). Low‐ and middle income countries such as China and India are expected to experience a rapid rise in population ageing, compared to Western Europe (United, 2017). Currently, over two thirds of people over 65 years of age are living with multi‐morbidities (Banerjee, 2015). When combined with parallel increases in disparities to health care and broader determinants of health (Sadana, Blas, Budhwani, Koller, & Paraje, 2016), there are major implications for health and social care systems (Beard et al., 2016; Chatterji, Byles, Cutler, Seeman, & Verdes, 2015; Prince Martin et al., 2015). While many nations are becoming wealthy with the influx of global socioeconomic developments, many countries, especially low‐ and middle income countries, have experienced increasing health and social disparities, especially among older adults (WHO, 2015). Older adults with the greatest health needs are also often those with the fewest resources to support them (Beard et al., 2016). For example, older adults in low‐ and middle income countries have poor access to assistive technologies and medical devices, as a result of a confluence of factors that affect the availability of these products in local markets including affordability and appropriateness, which can influence their availability, accessibility, and integration into health and social systems (Garçon et al., 2016; Marasinghe, Lapitan, & Ross, 2015). Furthermore, the privatization of health and social services becomes a barrier to quality of care if costs impact access to appropriate and timely care for older adults. Functional ability is complex and comprises an individual’s intrinsic capacity and people's interaction with their environment, including environmental characteristics that enable people to be and do what they value (Cesari et al., 2018; WHO, 2015). The WHO considers intrinsic capacity to include the physical and mental capacities of a person. Likewise, the environment defined by the WHO includes all factors in the extrinsic world that form the context of an individual’s life. For example, the home, community and society are included alongside the built environment, interpersonal relationships, attitudes, values, health and social policies, and the systems that support individuals and services (WHO, 2015).The Priority Assistive Products List of essential assistive devices that includes wheelchairs, pill organizers, hearing aids, and other essential items for many older people and people with disabilities to be able to live a healthy and dignified life and mitigate declines in intrinsic capacity (World, 2016).

The accumulation of exposures and environmental influences throughout the life course can influence the development of different risk factors that lead to chronic diseases, injuries, or other age‐related issues that contribute to declines in intrinsic capacities. Without a supportive environment, whether social or built, this will result in diminished functional ability. The gradual decline in intrinsic capacities as some people age can require increased health and social care services from informal (i.e., family or friends) and formal caregivers (i.e., health professionals). Increased care needs lead to increased burden on families, stress for older adults, and costs to society. For this reason, efforts to deliver cost efficient, effective interventions that optimize functional ability at any level of intrinsic capacity, is critical for older adults. Health and social care interventions (including assistive health technologies), and related systems, services and policies may include technological tools and devices and provision of health and social supports in the home.

While it is important to offer home‐based supports that promote functional ability, we need to be mindful that existing health inequities may be worsened (Sadana et al., 2016; Gottlieb & Alderwick, 2019). For example, if health and social services are provided privately and not covered by the health system or health insurance, all individuals will not have the same opportunities to achieve optimal health. Furthermore, the diagnosis, management and treatment of chronic health conditions in older adults can be prone to age‐based, unconscious bias and explicit discrimination, which can lead to less than optimal care (Cherubini, Corsonello, & Lattanzio, 2012; Drury, Abrams, Swift Hannah, Lamont Ruth, & Gerocova, 2017). Likewise, age‐based bias is seen in research on conditions that affect older adults such as stroke and osteoarthritis, with the median age of participants over 10 years younger than the typical patient (Gaynor, Geoghegan, & O'Neill, 2014; Liberopoulos, Trikalinos Nikolaos, & Ioannidis John, 2009).

### 1.2 The intervention

#### 1.2.1 Why it is important to develop the evidence and gap maps

An estimated >85% of research investment is wasted (Chalmers & Glasziou, 2009), some of which could be avoided by prioritizing research, including rigorous evaluation of existing evidence using systematic reviews (SR) prior to funding or carrying out new research (Chalmers et al., 2014). An evidence and gap maps (EGM) is a decision making and research prioritization tool that highlights gaps in research to inform strategic health and social policy, program and research priorities (Snilstveit, Vojtkova, Bhavsar, & Gaarder, 2013). EGMs can be used to avoid needless duplication, and can also be used to identify where sufficient, high quality evidence from systematic reviews and randomized trials are available as a basis for decisions or where sufficient studies are available for knowledge synthesis (Snilstveit, Vojtkova, Bhavsar, Stevenson, & Gaarder, 2016).

This EGM is important to inform policy and research prioritization. It is aligned with the WHO Strategy and Action Plan on Ageing and Health 2016–2020,. At the sixty‐ninth World Health Assembly in May 2016, the World Health Organization (2016) launched and received endorsement from all 193 member states for the WHO Strategy and Action Plan on Ageing and Health 2016‐2020. This plan outlined five strategic objectives: (a) commitment to action on healthy ageing in every country; (b) developing age‐friendly environments; (c) aligning health systems to the needs of older populations; (d) developing sustainable and equitable systems for providing long‐term care; (e) improving measurement, monitoring and research on healthy active ageing. The WHO aims to meet these by implementing evidence‐based actions to maximize functional ability of every individual (World, 2016). In this way the process of “optimizing opportunities for health, participation and security will enhance the quality of life as people age” (WHO, 2015). This EGM is relevant to the first objective – a commitment to action on healthy ageing in every country. Furthermore, our objectives align with the United Nations Sustainable Development Goals and the objectives of the UN High Level meeting on preventing and controlling non‐communicable diseases (United, 2018, 2019).

We will take a health systems perspective to extend the focus from health care to include social care and technological interventions. The evidence will be presented in terms of functional ability. We will also consider determinants of health inequity. The proposed EGM will consider the multi‐faceted and complex nature of functional ability and the various mechanisms (e.g., services, products, and individuals) involved in supporting functional ability among ageing adults.

We have a broad range of intended user groups including practitioners, researchers, policy/decision‐makers, and the public. We have also included three stakeholder groups as authors and advisory members: the WHO Department of Ageing and Life Course (RS), Cochrane Global Ageing (JTC) and the Campbell Ageing group (JTC). Each of these intended users are included as authors on the EGM and have participated in defining the intervention and outcome framework. The EGM will be used to identify the best quality evidence to guide decision making in this area. It will also help to identify gaps in the evidence base both in terms of evidence syntheses and primary research and thereby facilitate the prioritization of topics for further research.

Currently, no EGMs exist that identify and assess the available evidence on health, social care, and technological interventions to improve functional ability among older adults.

## 2 OBJECTIVES

The objectives are to:

- Identify available systematic reviews and randomized trials.
- Identify areas where systematic reviews are needed.
- Identify gaps in evidence where further primary research is needed.
- Assess equity considerations in available systematic reviews and randomized trials.
- Assess gaps and evidence related to health equity.

## 3 METHODS

### 3.1 Evidence and gap map: definition and purpose

We will adapt evidence gap map methods from various key papers (Bragge et al., 2011; Lum, Koper Christopher, & Telep Cody, 2011; Snilstveit et al., 2013, 2016) and will adopt a five‐stage process:

- Define a framework.
- Identify the available evidence.
- Appraise the quality of the evidence.
- Extract, code and summarize the data that relate to the objectives.
- Visualization and presentation of the findings in a user‐friendly manner.

We will use the Campbell Collaboration mapping tool developed by the EPPI‐Centre (Eppi‐Centre, 2019) to display identified studies using the framework described below.

### 3.2 Framework development and scope

The framework was developed following a meeting with methodologists, practitioners, decision‐makers and consumers at the Cochrane Colloquium during the 2017 Global Evidence Summit. Meeting participants suggested using the International Classification of Functioning (ICF) framework to define the intervention focus for this EGM as well as the outcomes. We further defined the scope of the framework in consultation with our research team which includes input from the public (AL), practitioners (LS, PT, KP, JN, ET, PW), information scientist (MR), policy‐makers (RS, HS) and researchers (VW, SM, JTC, MGC, EK, BS, AS, WZ).

The EGM framework will inform the inclusion and exclusion criteria. Key dimensions of the framework are listed below. We will follow the standard EGM framework as a matrix where the rows are intervention domains, and the columns are outcome categories. The interventions and outcomes will have sub‐categories.

Maintaining autonomy and independence, especially being able to make their own choices and decisions, are important for older adults in all settings (Hillcoat‐Nalletamby, 2014; Plath, 2009; Welford, Murphy, Rodgers, & Frauenlob, 2012). The concept of home is defined broadly, as the place of dwelling in which older adults seek to maintain their autonomy. This can include residential homes, long‐term care facilities, hospices, nursing homes and any non‐acute care places of residence. This EGM will identify health and social support services as well as technological interventions that optimize functional ability among older adults by systematically collecting, identifying, and mapping available evidence. In order to reflect the most relevant interventions and outcomes associated with supporting functional ability among older adults, we adapted the ICF framework and (WHO, 2001; Sadana & Posarac, 2018). The ICF is a comprehensive framework used by the WHO to measure health and disability at both individual and population levels. The WHO is also using the ICF as the basis to operationalize the measurement of intrinsic capacities, functional ability, and enabling environments (Sadana & Posarac, 2018)

Given the importance of the home setting in optimizing functional ability in older adults and in consultation with our advisory group, we are interested in technological interventions that support mobility at home (e.g., walking devices, ramps), social care services (e.g., homemaking, personal care) and health care services (e.g., covering promotive, preventive, treatment, rehabilitative, whether long‐term, provided by family physician visits or other health workers) delivered in the home setting.

### 3.3 Stakeholder engagement

We have created an Advisory Group comprised of methodologists, physicians (and other health‐care professionals), consumers and researchers with expertize in assistive health technology, healthy ageing, long‐term care, rehabilitation, disability, memory, and cognitive impairment. We held an exploratory meeting to invite feedback on the development of our EGM framework at the Global Evidence Summit in Cape Town, September 2017. The participants included family practitioners, geriatricians, social workers, and methodologists. We also held a seminar at the Bruyère Research Institute Grand Rounds (October 26, 2017) with family practitioner‐researchers, where participants provided feedback on the intervention‐outcome framework. Our decision to focus on the selected intervention categories was also informed by public engagement through our public representative (AL). Our central team (VW, TH, SM, PB, and CM) plans to meet at least once a month to discuss the direction and scope of the EGM.

### 3.4 Conceptual framework

**Figure 1:**
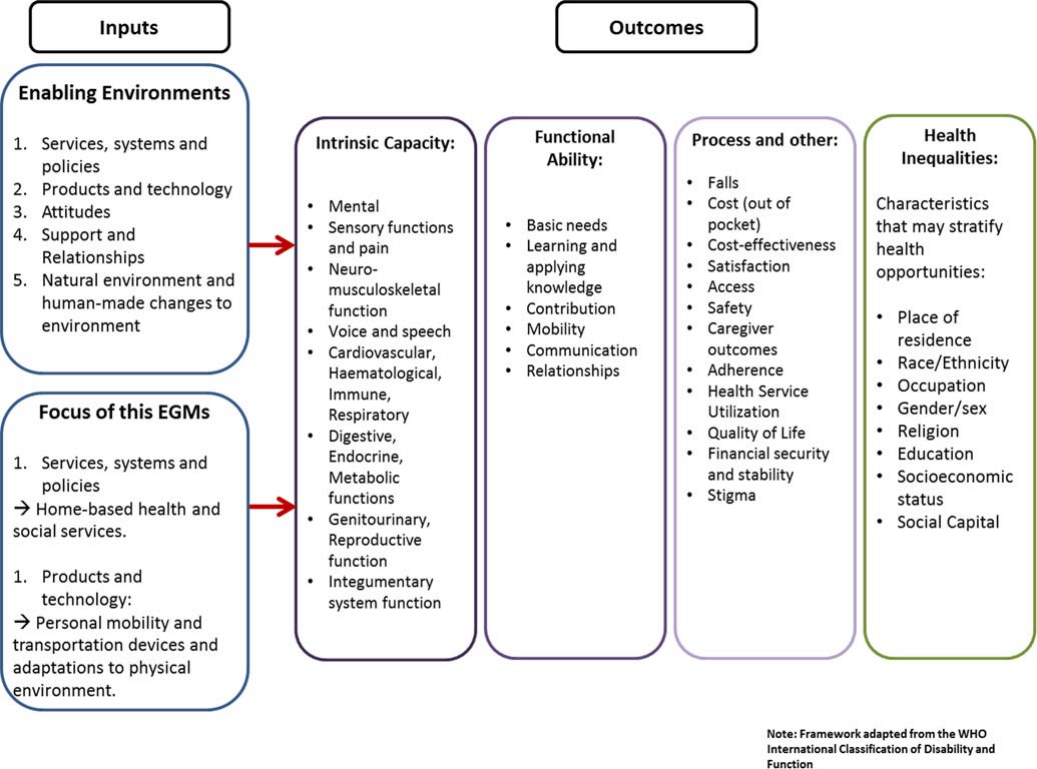
Conceptual framework [Color figure can be viewed at wileyonlinelibrary.com]

Figure 1 below demonstrates the conceptual framework through which the inputs lead to the intended outcomes. A person's intrinsic capacity is dependent on their health characteristics (e.g., health‐related behaviors, disease, or injuries), genetic inheritance, and personal characteristics (e.g., sex/gender or ethnicity). However, the extent to which an individual accomplishes what they value is also dependent on functional ability and their interactions with the environment, Enabling environments (i.e., services, systems and policies, and products and technology), when implemented within a home context, can influence outcomes such as improved neuro‐musculoskeletal functioning, through the use of an external aid, assistance by another person or improvement in the built environment. Supportive environments can strongly influence functional ability in the home among older adults. We also included health inequalities as an outcome of interest because we are aware that certain characteristics may stratify or impact health opportunities and outcome, such as socioeconomic status or place of residence.

We will review and update our conceptual framework as we develop the map.

## 4 DIMENSIONS

### 4.1 Types of study design

We will include randomized trials and systematic reviews of both randomized and nonrandomized trials that meet our inclusion criteria. We define a systematic review according to the PRISMA definition, which encompasses articles that specifically stated methods used to identify studies (i.e., a search strategy), strategies for study selection (e.g., eligibility criteria and selection process) and explicitly detailed methods of synthesis (Moher et al., 2015). This study design, as defined by PRISMA, uses a transparent and an a priori methodology in order to ensure rigor.

We will exclude systematic reviews of predictive factors, prognostic and diagnostic studies, and studies that primarily analyze implementation, barriers and facilitators to effectiveness (Snilstveit et al., 2013).

Since the aim of the EGM is to inform priorities for systematic reviews and knowledge exchange activities, we will also include on‐going systematic reviews and randomized trials. We will also include studies published in gray literature such as reports, dissertations, and conference abstracts.

We do not plan to include qualitative research.

### 4.2 Types of intervention/problem

We will contextualize interventions according to the ICF categorization of environmental factors. This will be further divided into:

1. Products and technology related to mobility: The ICF provides a very comprehensive list of eligible interventions. We will specifically examine section e1201 and e155 from the ICF that comprises assistive products and technology for personal indoor and outdoor mobility and transportation as well as design, construction, and building products and technology of buildings for private use. This includes products such as wheelchairs, walking devices, transfer devices, and ramps.
2. Health and social services, systems and policies: While we recognize that systems and policies can have an impact on the individual, we will specifically focus on sections e5750 and e5800, which includes health and social support services provided at home such as homemaking, personal care, health care professional home visits, or long‐term care.

We decided to limit the scope of the ICF framework due to feasibility. Specifically, we will exclude studies of pharmacological interventions, therapies, telemedicine or telecare, educational programs, and any hospital‐based programs. We will also exclude any studies that examine caregiver support services exclusively without evaluating outcomes related to older adults. A comprehensive list of interventions in each category may be found in Table 1.

**Table 1:** Interventions framework (based on the ICF)

Intervention category	Focus	Definition	Specific examples
**Services, Systems and Policies**	**e575** General social support services, systems and policies	Services, systems and policies aimed at providing support to those requiring assistance in areas such as shopping, housework, transport, child care, self‐care and care of others, in order to function more fully in society.	e5750 General social support servicesServices and programs aimed at providing social support to people who, because of age, poverty, unemployment, health condition or disability, require public assistance in the areas of shopping, housework, transport, self‐care and care of others, in order to function more fully in society.
		Exclusions: social security services, systems and policies (e570);	
		personal care providers and personal assistants (e340); health services, systems and policies (e580)	
	**e580** Health services, systems and policies		e5800 Health services
		Services, systems and policies for preventing and treating health problems, providing medical rehabilitation and promoting a healthy lifestyle. Exclusions: general social support services, systems and policies	Services and programmes at a local, community, regional, state or national level, aimed at delivering interventions to individuals for their physical, psychological and social well‐being, such as health promotion and disease prevention services, primary care services, acute care, rehabilitation and long‐term care services; services that are publicly or privately funded, delivered on a short‐term, long‐term, periodic or onetime basis, in a variety of service settings such as community, home‐based, school and work settings, general hospitals, specialty hospitals, clinics, and residential and non‐residential
**Products and Technology**	**e120** Products and technology for personal indoor and outdoor mobility and transportation	Equipment, products and technologies used by people in activities of moving inside and outside buildings, including those adapted or specially designed, located in, on or near the person using them.Inclusions: general and assistive products and technology for personal indoor and outdoor mobility and transportation	e1201 Assistive products and technology for personal indoor and outdoor mobility and transportation.Adapted or specially designed equipment, products and technologies that assist people to move inside and outside buildings, such as walking devices (such as canes or crutches), special cars and vans, adaptations to vehicles, wheelchairs, scooters and transfer devices.
	**e155** Design, construction and building products and technology of buildings for private use	Products and technology that constitute an individual's indoor and outdoor human‐made environment that is planned, designed and constructed for private use (e.g. home, dwelling), including those adapted or specially designed. Inclusions: entry and exits, facilities and routing	e1550 Design, construction and building products and technology for entering and exiting of buildings for private use Products and technology of entry and exit from the human‐made environment that is planned, designed and constructed for private use, such as entries and exits to private homes, portable and stationary ramps, power‐assisted doors, lever door handles and level door thresholds.

### 4.3 Types of population (as applicable)

This EGM will focus on adults over the age of 60 years, using the United Nations cut off for older or elderly individuals (United, 2015), and particularly those over 75 years of age. Studies and reviews will be included if at least 50% of the sample population is greater than 60 years old.

### 4.4 Types of outcome measures (as applicable)

We will map the evidence on outcomes that fall into one of the following ICF (WHO, 2001). adapted categories: impairments to body functions and structures (expressed as intrinsic capacities), and functional ability. We will also include process and other outcomes that may also have an effect on a particular outcome. We will consider health inequities by examining environmental and personal attributes that may stratify health opportunities and outcomes, using the PROGRESS framework (O'Neill et al., 2014). Our outcomes framework is provided in Table 3.

**Table 2:** Search strategy for MEDLINE

Category	Terms
Population	1 exp Aged/pc, px, rh [Prevention & Control, Psychology, Rehabilitation] (8053)
	2 "Aged, 80 and over"/ (806254)
	3 Frail Elderly/ (9588)
	4 elderly.ti,ab. (219354)
	5 older people.ti,ab. (23442)
	6 older adult*.ti,ab. (61366)
	7 older men.ti,ab. (7857)
	8 older women.ti,ab. (12791)
	9 old* age*.ti,ab. (65408)
	10 pensioners.ti,ab. (793)
	11 retirement.ti,ab. (11779)
	12 "end of life".ti,ab. (18653)
	13 (Resident* and (old* or home* or retirement or nursing)).ti,ab. (38765)
	14 geriatric*.ti,ab. (41516)
	15 (veteran* and (old* or home* or retire*)).ti,ab. (5047)
	16 or/1‐15 (1121318)
	17 exp Self‐Help Devices/ (10537)
	18 exp Orthopedic Equipment/ (92047)
Intervention	19 assistive devices.ti,ab. (1494)
	20 assistive equipment.ti,ab. (39)
	21 mobility equipment.ti,ab. (20)
	22 mobility device*.ti,ab. (311)
	23 mobility aid*.ti,ab. (276)
	24 motility.ti,ab. (85101)
	25 (walking adj2 (device* or aid* or equipment)).ti,ab. (1248)
	26 cane*.ti,ab. (5734)
	27 crutches.ti,ab. (1155)
	28 walking stick*.ti,ab. (202)
	29 (Adapt* adj3 (cars or transport or vehicles)).ti,ab. (506)
	30 (Adapt* adj3 (home* or house*)).ti,ab. (1545)
	31 Wheelchair*.ti,ab. (6462)
	32 exp Bathroom Equipment/ (10)
	33 scooter*.ti,ab. (368)
	34 transfer device*.ti,ab. (231)
	35 (communication adj (aid* or device*)).ti,ab. (858)
	36 exp Optical devices/ (88276)
	37 Hearing aids/ (7984)
	38 eyeglasses.ti,ab. (683)
	39 glasses.ti,ab. (10746)
	40 spectacles.ti,ab. (2316)
	41 hearing device*.ti,ab. (512)
	42 hearing aid*.ti,ab. (8346)
	43 vision aid*.ti,ab. (364)
	44 ((Adapt* or adjust*) adj3 (door* or entry or exit)).ti,ab. (239)
	45 Stair lift*.ti,ab. (2)
	46 stair climbing.ti,ab. (1444)
	47 stairs.ti,ab. (2902)
	48 stair rails.ti,ab. (2)
	49 shallow steps.ti,ab. (0)
	50 (ramp or ramps).ti,ab. (7094)
	51 Home Care Services/ (31353)
	52 home care service*.ti,ab. (1605)
	53 home support service*.ti,ab. (59)
	54 home visit*.ti,ab. (7662)
	55 community services.ti,ab. (2375)
	56 shopping.ti,ab. (3322)
	57 house help.ti,ab. (1)
	58 home help.ti,ab. (411)
	59 (food adj (preparation or assistance or help or service or delivery)).ti,ab. (3932)
	60 (meal* adj3 (provision or assistance or help or service* or preparation or delivery)).ti,ab. (1137)
	61 homemaking.ti,ab. (109)
	62 housekeeping.ti,ab. (8477)
	63 ((household or ktichen or routine) adj (jobs or tasks or chores)).ti,ab. (888)
	64 bathing.ti,ab. (9571)
	65 grooming.ti,ab. (5015)
	66 personal hygiene.ti,ab. (1847)
	67 toileting.ti,ab. (857)
	68 foot care.ti,ab. (1270)
	69 (medication adj2 reminders).ti,ab. (147)
	70 (kitchen or bathroom or bedroom).ti,ab. (5694)
	71 or/17‐70 (400411)
Outcomes	72 exp "Activities of Daily Living"/ (63476)
	73 Human Activities/ (2170)
	74 Automobile Driving/ (17307)
	75 Leisure Activities/ (7897)
	76 "activities of daily living".ti,ab. (22139)
	77 "quality of life".ti,ab. (229433)
	78 "Quality of Life"/ (164112)
	79 independence.ti,ab. (36023)
	80 wellbeing.ti,ab. (11362)
	82 social participation.ti,ab. (2177)
	83 happiness.ti,ab. (5642)
	84 happier.ti,ab. (734)
	85 mental health.ti,ab. (116393)
	86 functional ability.ti,ab. (4311)
	87 depression.ti,ab. (289365)
	88 cognitive.ti,ab. (296200)
	89 sensory function*.ti,ab. (3884)
	90 pain.ti,ab. (543562)
	91 distress.ti,ab. (97018)
	92 vitality.ti,ab. (10533)
	93 energy.ti,ab. (544017)
	94 fatigue.ti,ab. (80717)
	95 tiredness.ti,ab. (3430)
	96 self care.ti,ab. (14789)
	97 self efficacy.ti,ab. (21966)
	98 mobility.ti,ab. (123516)
	99 community life.ti,ab. (457)
	100 security.ti,ab. (38430)
	101 relationships.ti,ab. (322577)
	102 satisfaction.ti,ab. (113208)
	103 adherence.ti,ab. (98155)
	104 reablement.ti,ab. (49)
	105 institutionali?ation.ti,ab. (4370)106 or/72‐105 (2682926)
	107 systematic*.ti,ab. (374866)
	108 (meta‐analysis or metaanalysis).ti,ab. (112568)
	109 (review* and (literature or studies or trials)).ab. (693115)
	110 review.ti. (393065)
	111 (evidence adj2 synthesi*).ti,ab. (5932)
	112 overview.ti,ab. (139107)
	113 pubmed.ab. (82182)
	114 medline.ab. (94705)
	115 or/107‐114 (1336239)
Study Design	116 randomized controlled trial.pt. (464926)
	117 controlled clinical trial.pt. (92516)
	118 randomized.ti,ab. (448898)
	119 randomly.ab. (294026)
	120 trial.ti,ab. (509010)
	121 groups.ab. (1815046)
	122 usual care.ab. (13020)
	123 or/116‐122 (2634734)
	124 115 or 123 (3780045)
	125 16 and 71 and 106 and 124 (3987)

**Table 3:** Outcomes Framework

Outcome category	Measure/construct
Intrinsic Capacity	• Mental
	• Sensory functions and pain
	• Neuro‐musculoskeletal function
	• Voice and speech
	• Cardiovascular, Haematological, Immune, Respiratory
	• Digestive, Endocrine, Metabolic functions
	• Genitourinary, Reproductive function
	• Integumentary system function
Functional Ability	• Basic needs
	• Learning and applying knowledge
	• Contribution
	• Mobility
	• Communication
	• Relationships
Process and other	• Falls
	• Cost (out of pocket)
	• Cost‐effectiveness
	• Satisfaction
	• Access
	• Safety
	• Caregiver outcomes
	• Adherence
	• Health Service Utilization
	• Quality of Life
	• Financial security and stability
	• Stigma
Health Inequalities	• Place of residence
	• Race/Ethnicity
	• Occupation
	• Gender/sex
	• Religion
	• Education
	• Socioeconomic status
	• Social Capital

Intrinsic capacity will consist of mental (e.g., depression, sleep, vitality); sensory functions and pain (e.g., vision, hearing); neuro‐musculoskeletal function (e.g., gait, balance); voice and speech (e.g., articulation); cardiovascular, hematological, immune, respiratory system function (e.g., blood pressure, respiration); digestive, endocrine, metabolic functions (e.g., thyroid, glucose); genitourinary and reproductive function (e.g., bladder control); and integumentary system function (e.g., skin, nails).

Functional ability will consist of the following constructs: basic needs (e.g., self‐care, acquisition of goods and services); learning and applying knowledge (e.g., applying knowledge); contribution (e.g., community life, employment); mobility (e.g., walking); relationships (e.g., interpersonal interactions); and communication (e.g., language).

Process and other outcomes will include cost (out of pocket), cost‐effectiveness, falls, satisfaction of older adult, safety, caregiver outcomes, adherence, health service utilization, quality of life, financial security, access, and stigma. Access is a multi‐faceted concept and can be understood as the opportunity or ease with which consumers or communities are able to use appropriate services in proportion to their needs (Daniels, 1982; Whitehead, 1992). The concept of access will include: acceptability, approachability, availability and accommodation, affordability and appropriateness (Levesque, Harris Mark, & Russell, 2013).

We will use the PROGRESS framework to identify studies that measured effects of interventions by gender or other health inequalities.

### 4.5 Other eligibility criteria

#### 4.5.1 Types of settings

We will include interventions in the home setting for older adults. We will define home as an individual’s place of residence that can include residential homes, apartments, long‐term care facilities, hospices, nursing homes and any nonacute care places of residence. Studies of mixed settings will be included as long as the intervention takes place in the home setting at least 50% of the time. We will code the settings so that the evidence can be filtered according to setting.

Acute and sub‐acute hospital and convalescent care settings will be excluded (e.g., geriatric rehabilitation in subacute care).

### 4.6 Search methods and sources

We will develop and pilot a search strategy (with a selection of studies that met our inclusion criteria) with the guidance of an information scientist (MR). This search will comprise of the medical and health databases ( MEDLINE (via OvidSp), EMBASE (via OvidSp), Cochrane Database of Systematic Reviews, CENTRAL, CINAHL (Via EBSCOhost), PsychINFO (via OvidSp) and AgeLine (via EBSCOhost) and databases relevant to social care and social policy (Campbell Library, ASSIA (via ProQuest), Social Science Citation Index (via Web of Science) and Social Policy and Practive (via OvidSp). See Table 2 for full search strategy.

We will search for relevant trials and systematic reviews in the gray literature via ProQuest Theses and Dissertation Global and via Conference Proceedings Citation Index. We will also search for relevant unpublished studies via relevant international organizations (e.g., Help Age, World Health Organization, and Institute for Research on Public Policy).

Ongoing systematic reviews will be identified by searching for protocols in PROSPERO and the Cochrane and Campbell libraries as well as on the open science framework (https://osf.io/). Ongoing randomized trials will be searched in ClinicalTrials.gov and the WHO International Clinical Trials Registry Platform.

## 5 ANALYSIS AND PRESENTATION

### 5.1 Report structure

The EGM report will include the following sections: executive summary, background, methods, results, and conclusion. The executive summary will summarize the report, providing the main findings and implications for future policy planning and research. The background will provide a comprehensive description of the burden of illness and disability among aging older adults and the impact on functional ability and state the objectives of the EGM. We will also describe the scope by defining the intervention and outcomes framework, and theory of change (supplemented by a figure).

Description of the methods will include a definition of the data sources and methods of searching, the inclusion and exclusion criteria, study selection, quality appraisal, and data extraction methods and the approach to presentation/visualization. This section will provide a table in‐text showing one full search from a database as well as a PRISMA flow chart. An appendix will provide full search strategies used for each database, including any restrictions and filters used.

The results section will present the number, type, and quality of studies retrieved for the main intervention categories, namely, products and technology related to mobility, and health and social services at home. We will also present information about how health equity has been considered in studies, provide an overview of major evidence gaps based on our framework, and list any limitations. The conclusion will provide implications for researchers, decision‐makers and policymakers on the evidence base in this area and the key areas for the commissioning of future research. We will also include implications for researchers considering conducting an EGM.

Tables and figures we will include:

- Figure: Conceptual map/theory of change.
- Figure: PRISMA flow chartTable: number of studies by study design.
- Table: Number of studies by intervention and subcategories.
- Table: Number of studies by population.

### 5.2 Filters for presentation

We will present results as a matrix of interventions (rows) and outcomes (columns) and assess the availability of evidence across the additional filters of age groups, conditions, equity categories (i.e., PROGRESS) and setting.

### 5.3 Dependency

When there are multiple reports for a single study, we will treat them as one study. We acknowledge that SRs are likely to include the RCTs in the map and there may be more than one SR which includes the same RCT. All relevant randomized trials will be included regardless of whether or not it is included in a systematic review.

## 6 DATA COLLECTION AND ANALYSIS

### 6.1 Screening and study selection

Two reviewers will independently screen the titles and abstracts of all retrieved articles. Title and abstracts will be screened on the basis of intervention, study design, and population, and not on the basis of outcome. Full‐texts of potentially eligible studies will then be retrieved and screened. The reviewers will compare the results, and any conflicts will be resolved through discussion or by a third reviewer. We will not contact authors of studies or reviews for missing information.

### 6.2 Data extraction and management

Two reviewers will independently extract data on published and ongoing systematic reviews and randomized trials related to the population, intervention, comparison, and outcomes. Our coding categories (Appendix) for data extraction will be based on our intervention/outcomes framework. In addition, we will collect details on characteristics that may be of interest to decision makers as filters for the evidence ‐ the country of the study (using WHO regions as well as the World Bank country classifications by income), age group (e.g., <65 years, >65 years, etc. ), health conditions (e.g., communicable, noncommunicable), study design (e.g., RCT or systematic review) and setting (e.g., residential home, independent living, assisted living, or long term care).

We will also collect details, if reported, on health equity as defined according to the PROGRESS framework ‐ place of residence, race/ethnicity/language/culture, occupation, gender/sex, religion, education, socioeconomic status, social capital and other characteristics associated with disadvantage and vulnerability such as sexual orientation, age and disability (O'Neill et al., 2014). Furthermore, we will examine whether studies assessed the effects of the intervention by gender or any other characteristic of health inequality such as socioeconomic status. For systematic reviews, we will report equity characteristics as described, and will not go back to included primary studies for more details.

### 6.3 Tools for assessing risk of bias/study quality of included reviews

Since systematic reviews are often used for decision making, we will appraise the quality of systematic reviews with AMSTAR‐2 (Shea, Reeves, & Wells, 2017) in duplicate for 10% of eligible studies. Kappa statistics will also be used to check agreement for each item. If the agreement is over 80%, we will proceed with single data extraction with verification by a second reviewer for the remainder of studies.

The quality of randomized trials is not usually assessed in EGMs since the purpose is to identify the randomized trials available, and not to make decisions based on single trials. As such, we will not assess the quality of randomized trials (Snilstveit, Bhatia, Rankin, & Leach, 2017).

### 6.4 Methods for mapping

We plan to use the EPPI‐Reviewer 4 software to generate the map (EPPI‐Centre, 2019).

## AUTHOR CONTRIBUTIONS

- Content: T. H., V. W., H. S., S. M., provide content expertise in rehabilitation, assistive devices and memory and cognitive impairment. C. M. and L. S. also have expertise in ageing and rehabilitation. EK has expertise in built environments and in healthy aging. L. S., J. B., W. Z., J. C. T., A. L., J. N., P. T., P. W., and B. S. provided content expertise on classifying outcomes and interventions, and will provide critical comments on final manuscript.
- EGM methods: V. W., A. S., S. M., T. H., K. P. and E. K. are experts in systematic review methods
- Information retrieval: M. R. is an information specialist with experience in designing searches for systematic reviews.

## DECLARATIONS OF INTEREST

V. W. is Editor in Chief of the Campbell Collaboration.

Johan Borg is employed as research manager at a commercial assistive technology company that may have an interest in the results or conclusions of this review.

The rest of the authors have no conflicts of interest with respect to the content of the EGM.

## PRELIMINARY TIMEFRAME

Approximate date for submission of the EGM: October 2019

Please note this should be no longer than one year after protocol approval.

## PLANS FOR UPDATING THE EGM

Vivian Welch, Tracey Howe and Sue Marcus, as directors of Cochrane Global Ageing, have an interest in continuing to update this EGM. Frequency of updating will depend on availability of resources to do so.

## SOURCES OF SUPPORT

### Internal sources

- No sources of support provided

### External sources

This EGM is funded by the Canadian Institutes of Health Research (Funding reference numbers: 400672; 385789).

## APPENDIX 1

**1 Coding Tool**

**Table:**

Category		Answer
Geographical information	WHO Regions	‐ South Asia
		‐ Sub‐Saharan Africa
		‐ East Asia and Pacific
		‐ Europe and Central Asia
		‐ Latin America and Caribbean
		‐ Middle East and North Africa
		‐ North America
	World Bank Region (2019 FY)	‐ Low income economies
		‐ Lower Middle income economies
		‐ Upper Middle income economies
		‐ High income economies
Study design	Design	‐ Systematic reviews
		‐ RCT
	Publication status	‐ Complete
		‐ On‐going (e.g. Protocols)
Population	Age Group	‐ Includes<65 years
		‐ Includes>65 years
		‐ Includes>75 years
		‐ Includes>85 years
	Sex/Gender	‐ Includes LGBTQ2 + ‐ Proportion of females included in study
Health Conditions		‐ Communicable Disease
		‐ Non‐communicable disease
		‐ Injury
		‐ Discharge from hospital
		‐ End‐of‐life
		‐ Physical Frailty
		‐ Social Frailty
		‐ Care Dependent
Intervention	General Social support services, systems and policies	‐ Homemaking
		‐ Personal Care
		‐ Transportation
		‐ Family/Caregiver support
		‐ Friendly visits
	Health services, systems and policies	‐ General health services for disease prevention
		‐ Health promotion services
		‐ Rehabilitation Services
		‐ Long‐term care services
		‐ Visiting Health Professionals
		‐ Visiting Lay care providers
	Products and Technology	‐ Personal mobility and transportation devices
		‐ Adaptations to physical environment
Outcome	Intrinsic Capacity	‐ Mental
		‐ Sensory functions and pain
		‐ Neuro‐musculoskeletal function
		‐ Voice and speech
		‐ Cardiovascular, Haematological, Immune, Respiratory
		‐ Digestive, Endocrine, Metabolic functions
		‐ Genitourinary, Reproductive function
		‐ Integumentary system function
	Functional Ability	‐ Basic needs
		‐ Learning and applying knowledge
		‐ Contribution
		‐ Mobility
		‐ Communication
		‐ Relationships
	Process and other	‐ Falls
		‐ Cost (out of pocket)
		‐ Cost‐effectiveness
		‐ Satisfaction
		‐ Access
		‐ Safety
		‐ Caregiver outcomes
		‐ Adherence
		‐ Health Service Utilization
		‐ Quality of Life
		‐ Financial security and stability
		‐ Stigma
Setting		‐ Residential home/apartment
		‐ Long‐term care
		‐ Independent living
		‐ Assisted Living
Comparison		‐ Usual Care
‐ Other		
Systematic Review quality		‐ High
		‐ Moderate
		‐ Low
		‐ Critically Low
		‐ Protocol
PROGRESS		‐ Place of residence
		‐ Race/Ethnicity
		‐ Occupation
		‐ Gender/sex
		‐ Religion
		‐ Education
		‐ Socioeconomic status
		‐ Social Capital
Gender Inequalities	Is there an assessment of effects by sex/gender	‐ Yes
		‐ No
		‐ Planned but not reported
Other inequalities	Is there an assessment of effects by other characteristics, e.g. socioeconomic status, income, race/ethnicity, etc	‐ Yes
		‐ No
		‐ Planned but not reported
